# Paradoxical improvement of life quality in the COVID-19 era in psoriasis patients

**DOI:** 10.1371/journal.pone.0275293

**Published:** 2022-09-27

**Authors:** Katharina Boch, Detlef Zillikens, Ralf J. Ludwig, Diamant Thaçi

**Affiliations:** 1 Department of Dermatology, University of Lübeck, Lübeck, Germany; 2 Lübeck Institute of Experimental Dermatology, University of Lübeck, Lübeck, Germany; 3 Institute and Comprehensive Center for Inflammation Medicine, University of Lübeck, Lübeck, Germany; University of Hail, SAUDI ARABIA

## Abstract

**Background:**

Psoriasis is a chronic immune-mediated inflammatory disease. Beyond the physical dimensions, the disease has an extensive emotional and psychosocial effect on patients, influencing their quality of life, social life and interpersonal relationships. Thus patient-reported outcomes are a crucial instrument for the evaluation of disease burden. Navigating life in times of the COVID-19 pandemic is challenging, especially for persons suffering from chronic diseases. We here analyzed the impact of lockdown restrictions on psoriasis patients.

**Objective:**

To compare the Dermatology Life Quality Index (DLQI) before and during the COVID-19 pandemic of patients with psoriasis.

**Methods:**

Retrospective longitudinal analysis in adult patients with moderate to severe psoriasis undergoing biologic treatment between January 2020 and January 2021. DLQI, patient demographics, Psoriasis Area and Severity Index (PASI), and recent biologic treatment were recorded.

**Results:**

103 patients were identified, of whom 19 had additional psoriatic arthritis. Female (n = 29) and male (n = 74) patients were distributed 1 to 3. Median age of patients was 54 years (range 18–85). All patients received biologic systemic treatment: anti-IL-23 (n = 39), anti-IL-17A (n = 30), anti-IL-12/23 (n = 25), or anti-TNFα (n = 9). Comparing DLQI scores before the COVID-19 pandemic and under lockdown restriction showed improved DLQI scores over time. Further analysis displayed that patients mostly ticked “not relevant” on social activities during lockdown. Thus, the DLQI scores may be artificial improved and may not really reflect the actual disease burden.

**Conclusions:**

Psoriasis patients showed a contrary improvement of life quality despite harsh COVID-19 lockdown suggesting that DLQI should be modified when social life is restricted.

## Introduction

Psoriasis is a chronic immune-mediated inflammatory disease affecting the skin and is frequently associated with joint disorders (psoriatic arthritis) [1–3]. Furthermore, the psoriatic disease is leading to numerous comorbidities including metabolic syndrome, depression and cardiovascular diseases[1,2]. Affecting visible areas such as face, hands and nails, psoriasis may create stigmatization and result in a heavy emotional burden and extensive psychosocial effects affecting personal and social life as well as interpersonal relationships [4]. Furthermore, skin lesions often are accompanied by agonizing pruritus. Joint involvement frequently is associated with chronic pain, myalgia and movement restrictions. This additionally can lead to the reduction of psychological conditions and have a negative impact on well-being [4] Depending on the severity of disease and the affection of life quality, treatment is initiated [5–8]. Dermatology Life Quality Index (DLQI) is a questionnaire with 10 items, covering personal discomfort (itch, sore, pain or stinging) and perception (embarrassment, self-consciousness), but also personal interactions (partner, family, friends). Furthermore, the impact of the skin disease on mandatory duties (work/school, shopping, housekeeping, gardening), and social/leisure activities as well as sports are queried. Questions of impairment can be answered with very much (scored 3), a lot (scored 2), a little (scored 1), not at all (scored 0) or not relevant (scored 0). The final DLQI is calculated by summing the score of each question, the higher the score, the more quality of life is impaired. At the beginning of 2020, the human-to-human transmission of COVID-19 forced governments worldwide to implement restrictions on individual mobility and face-to-face interaction. These restrictive measures ranged from working-from-home to ‘shelter-in-place’, closure of schools and non-essential businesses. In Germany, there were massive restrictions in social life to keep people safe. To date, data on the effects of social restrictions under lockdown regulations during the COVID-19 pandemic on life quality and social life of psoriasis patients are lacking. Thus, the aim of this retrospective single-center cohort study was to longitudinal compare patient-reported outcomes before the COVID-19 era and under harsh measures initiated to contain the spread of the virus.

## Materials and methods

The current retrospective cohort study included selected patients diagnosed with moderate to severe psoriasis treated with biologics between January 1^st^_,_ 2020, and March 31^th^, 2021 at the Comprehensive Center for Inflammation Medicine, University of Lübeck, Lübeck, Germany. The study was approved by the institutional ethical committee in accordance with the Declaration of Helsinki. All patients have given written informed consent.

The electronic medical records of eligible patients were systematically reviewed. Psoriasis was diagnosed based on compatible clinical manifestation +/- confirmatory histology. Joint involvement was confirmed by clinical examination and radiologic findings at baseline. The following variables were retrieved: PASI^5^ and DLQI^6^ before onset of the pandemic (January 2020) and under social life restrictions due to due lockdown (January 2021) as well as the utilized systemic therapeutic modalities. DLQI questionnaires were handed out to the patients at their regular visits at the Comprehensive Center for Inflammation Medicine, University of Lübeck, Lübeck, Germany.

## Results and discussion

The current study included 103 patients with moderate to severe psoriasis with or w/o joint involvement, of whom 29 (28.2%) were females and 74 (71.8%) were males. The median (range) age of study participants was 54 (18–85) years. Psoriasis was the most frequently encountered manifestation (n = 84; 81.6%), whereas PsA was present in 19 (18.4%) patients (**Table 1**). The mean (SD) PASI score pre-COVID was 3.6 (5.6). Under lockdown restriction, mean (SD) PASI score was calculated 1.6 (2.31). Of note, all patients were under ongoing continuous biologic treatment at the time point of study inclusion, this may reflect the low PASI at baseline and only slight improvement over time. The majority of study participants (n = 39; 37.9%) underwent treatment with anti-IL-23. Thirty (29.1%) patients were managed by anti-IL-17A, 25 (24.3%) patients were under anti-IL-12/23 and 9 (8.7%) patients were treated with anti-TNFα as delineated in **Table 1**.

**Table 1:** Demographic and clinical characteristics.

Age at diagnosis; years	
Median (range)	54 (18–85)
**Sex, n (%)**	
Male	74 (71.8%)
Female	29 (28.2%)
Morphology of disease, n (%)*	
Psoriasis	84 (81.6%)
Psoriatic arthritis	19 (18.4%)
**Treatment, n (%)**	
anti-TNFα	9 (8.7%)
anti-IL-12/23	25 (24.3%)
anti-IL-17A	30 (29.1%)
anti-IL-23	39 (37.9%)
**Psoriasis Area and Severity Index (PASI)**	
PASI before pandemic, mean (SD)	3.6 (5.9)
PASI before pandemic, median (range)	1.8 (0–42.7)
PASI during lockdown, mean (SD)	1.6 (2.31)
PASI during lockdown, median (range)	1.0 (0–11.2)
**Dermatology Life Quality Index (DLQI)**	
DLQI before pandemic, mean (SD)	5.6 (7.38)
DLQI before pandemic, median (range)	2.0 (0–30)
DLQI during lockdown, mean (SD)	2.7 (5.11)
DLQI during lockdown, median (range)	1.0 (0–25)

**Abbreviations:** anti-TNFα, tumour necrosis factor α inhibitor; DLQI, Dermatology Life Quality Index; IL, Interleukin; n, number; PASI, Psoriasis Area and Severity Index; SD, standard deviation.

The longitudinal quality of life analysis of this cohort of psoriasis patients showed that the mean (SD) and median (range) DLQI scores shortly before the COVID-19 pandemic were 5.6 (7.38) and 2.0 (0–30), respectively. Whereas under lockdown restriction, a mean (SD) 2.7 (5.11) and a median (range) 1.0 (0–25) DLQI score was calculated (**Table 1**). Comparing DLQI scores shortly before the onset of the COVID-10 pandemic and under harsh lockdown restrictions demonstrated that life quality of most patients with psoriasis, contrary to what was expected, improved. Analyzing DLQI questionnaires on social activities during pandemic showed that questions concerning social activities like work/school, shopping, and social/leisure activities as well as and self-perception and personal interactions were answered with “not relevant” or “not at all” (**Table 2**). As this item is scored 0, this may lead to an artificial improvement of life quality measurements. This paradoxical improvement of DLQI during the COVID-19 pandemic suggests that DLQI should be modified when social life is restricted, as these scores evaluated under restrictions (with a lot “not relevant” responses) do not reflect the true extent of negative impact on life quality. This is in line with recently published observation which showed that DLQI improved due to “not relevant” responses [9]. Moreover, it was shown that the “not relevant” ticked box can have an impact on the treatment decisions [10]. This data underlines, the importance of adjusted validation of patient-reported outcome scores in pandemic or patient-individual situations [9–12].

**Table 2:** Dermatology Life Quality Index measurement of social activities.

n = 103	Covid-19 Lockdown
**Shopping, looking after home/garden n (%)**	
Very much	-
A lot	-
A little	-
Not at all	64 (62.1)
Not relevant	39 (37.4)
**Social/Leisure activities n (%)**	
Very much	-
A lot	15 (14.6)
A little	10 (9.7)
Not at all	31 (30.1)
Not relevant	47 (34.6)
**Sport, n (%)**	
Very much	-
A lot	-
A little	7 (6.7)
Not at all	57 (55.4)
Not relevant	39 (37.9)
**Working/Studying, n (%)**	
Very much	-
A lot	-
A little	11 (10.7)
Not at all	47 (45.6)
Not relevant	45 (43.7)
**Interaction (partner, close friends, relatives), n (%)**	
Very much	-
A lot	-
A little	-
Not at all	41 (39.8)
Not relevant	62 (60.2)

Abbreviations: n, number.

Limitations of the study include the relatively small number of patients and its retrospective nature. This may have resulted in some degree of selection bias, given that the patients were attending a tertiary referral center. Psoriasis treatment had been initiated prior to inclusion to this analysis.

## Conclusions

This retrospective cohort study revealed that DLQI paradoxically improved under social restrictions, increasing the awareness to life situation adjusted validation of DLQI scores. Our findings underscore the importance of individual evaluation of patient-reported outcomes in order to reflect the burden of the underlying chronic skin disease and not the impact of general life circumstances. Furthermore this data underlines the effects of “not relevant” answer on the validity of DLQI score.

## Supporting information

S1 Table Patient database. (DOCX)

S2 Table Dermatology Life Quality Index measurement of social activities during COVID-19 lockdown. (DOCX)

## References

[1] Griffiths CEM, Armstrong AW, Gudjonsson JE, Barker JNWN. Psoriasis. Lancet. 2021 Apr 3;397(10281):1301–1315. doi: 10.1016/S0140-6736(20)32549-6 33812489

[2] Griffiths CE, Barker JN. Pathogenesis and clinical features of psoriasis. Lancet. 2007 Jul 21;370(9583):263–271. doi: 10.1016/S0140-6736(07)61128-3 17658397

[3] Veale DJ, Fearon U. The pathogenesis of psoriatic arthritis. Lancet. 2018 Jun 2;391(10136):2273–2284. doi: 10.1016/S0140-6736(18)30830-4 29893226

[4] Mattei PL, Corey KC, Kimball AB. Psoriasis Area Severity Index (PASI) and the Dermatology Life Quality Index (DLQI): the correlation between disease severity and psychological burden in patients treated with biological therapies. J Eur Acad Dermatol Venereol. 2014 Mar;28(3):333–7. doi: 10.1111/jdv.12106 23425140

[5] Frediksson T, Pettersson U. Severe psoriasis–oral therapy with a new retinoid. Dermatologica. 1978;157:238–44. doi: 10.1159/000250839 357213

[6] Finlay AY, Khan GK. Dermatology Life Quality Index (DLQI): a simple practical measure for routine clinical use. Clin Exp Dermatol 1994; 19:210–216. doi: 10.1111/j.1365-2230.1994.tb01167.x 8033378

[7] Menter A, Strober BE, Kaplan DH, Kivelevitch D, Prater EF, Stoff B, et al. Joint AAD-NPF guidelines of care for the management and treatment of psoriasis with biologics. J Am Acad Dermatol. 2019 Apr;80(4):1029–1072. doi: 10.1016/j.jaad.2018.11.057 30772098

[8] Herédi E, Rencz F, Balogh O, Gulácsi L, Herszényi K, Holló P, et al. Exploring the relationship between EQ-5D, DLQI and PASI, and mapping EQ-5D utilities: a cross-sectional study in psoriasis from Hungary. Eur J Health Econ. 2014 May;15 Suppl 1:S111–9. doi: 10.1007/s10198-014-0600-x 24832842

[9] Kearney N, Hambly R, Alsharqi A, Kirby B. ’Not relevant’ responses in the era of COVID-19: are we underestimating Dermatology Life Quality Index values? Br J Dermatol. 2022 Jan;186(1):187–189. doi: 10.1111/bjd.20705 Epub 2021 Oct 21. 34427918 PMC8652704

[10] Rencz F, Poór AK, Péntek M, Holló P, Kárpáti S, Gulácsi L, et al. A detailed analysis of ’not relevant’ responses on the DLQI in psoriasis: potential biases in treatment decisions. J Eur Acad Dermatol Venereol. 2018 May;32(5):783–790. doi: 10.1111/jdv.14676 Epub 2017. 29114942

[11] Basra MK, Fenech R, Gatt RM, Salek MS, Finlay AY. The Dermatology Life Quality Index 1994–2007: a comprehensive review of validation data and clinical results. Br J Dermatol. 2008 Nov;159(5):997–1035. doi: 10.1111/j.1365-2133.2008.08832.x 18795920

[12] Hongbo Y, Thomas CL, Harrison MA, Salek MS, Finlay AY. Translating the science of quality of life into practice: What do dermatology life quality index scores mean? J Invest Dermatol. 2005 Oct;125(4):659–64. doi: 10.1111/j.0022-202X.2005.23621.x 16185263

