# Supplementary material for: Paradoxical improvement of life quality in the COVID-19 era in psoriasis patients
Source: PLoS One. 2022 Sep 27;17(9):e0275293. doi: 10.1371/journal.pone.0275293 (PMC9514635; doi:10.1371/journal.pone.0275293)
Supplement: S1 Table — (DOCX) [file pone.0275293.s001.docx]

**S1 Table. Patient database.**

| Patient | Sex | Age | Diagnosis | Substance | PASI pre COVID | DLQI pre COVID | PASI lockdown | DLQ lockdown |
| --- | --- | --- | --- | --- | --- | --- | --- | --- |
| #1 | f | 69 y | Pso/PsoA | anti-TNFα | 2 | 18 | 1 | 8 |
| #2 | f | 50 y | Pso/PsoA | anti-TNFα | 1 | 0 | 1 | 0 |
| #3 | f | 52 y | Pso/PsoA | anti-TNFα | 0.6 | 11 | 0 | 12 |
| #4 | m | 61 y | Pso/PsoA | anti-TNFα | 2.4 | 3 | 1 | 20 |
| #5 | m | 63 y | Pso/PsoA | anti-TNFα | 2 | 15 | 2.3 | 9 |
| #6 | m | 61 y | Pso/PsoA | anti-TNFα | 2 | 9 | 3 | 1 |
| #7 | f | 66 y | Pso | anti-IL-17A | 0 | 0 | 0 | 0 |
| #8 | f | 65 y | Pso | anti-IL-17A | 6 | 5 | 8 | 4 |
| #9 | m | 30 y | Pso | anti-IL-17A | 0 | 1 | 0 | 1 |
| #10 | m | 30 y | Pso | anti-IL-17A | 2 | 1 | 10 | 22 |
| #11 | m | 54 y | Pso | anti-IL-17A | 8.8 | 19 | 8 | 8 |
| #12 | m | 55 y | Pso | anti-IL-17A | 1 | 2 | 1.2 | 4 |
| #13 | m | 47 y | Pso | anti-IL-17A | 15 | 0 | 4.8 | 0 |
| #14 | m | 85 y | Pso | anti-IL-17A | 1.2 | 1 | 2.0 | 0 |
| #15 | m | 44 y | Pso | anti-IL-17A | 0 | 0 | 0 | 0 |
| #16 | m | 35 y | Pso | anti-IL-17A | 0 | 0 | 0 | 0 |
| #17 | m | 55 y | Pso | anti-IL-17A | 2.8 | 1 | 6.1 | 0 |
| #18 | m | 49 y | Pso | anti-IL-17A | 8.6 | 11 | 11.2 | 8 |
| #19 | f | 27 y | Pso | anti-IL-17A | 3.6 | 30 | 1 | 1 |
| #20 | m | 50 y | Pso | anti-IL-17A | 4.3 | 0 | 4.4 | 0 |
| #21 | m | 63 y | Pso | anti-IL-17A | 0 | 0 | 1 | 0 |
| #22 | m | 57 y | Pso | anti-IL-17A | 0 | 0 | 0 | 0 |
| #23 | m | 53 y | Pso | anti-IL-17A | 0 | 0 | 2.2 | 0 |
| #24 | m | 49 y | Pso | anti-IL-17A | 1.8 | 1 | 3 | 0 |
| #25 | m | 43 y | Pso | anti-IL-17A | 3 | 0 | 3.4 | 0 |
| #26 | f | 64 y | Pso/PsoA | anti-IL-17A | 0 | 0 | 1 | 3 |
| #27 | m | 56 y | Pso | anti-IL-17A | 0 | 0 | 0 | 0 |
| #28 | m | 69 y | Pso | anti-IL-17A | 2.4 | 2 | 1 | 0 |
| #29 | m | 50 y | Pso | anti-IL-17A | 0 | 2 | 3 | 1 |
| #30 | f | 39 y | Pso | anti-IL-17A | 1 | 0 | 0.6 | 0 |
| #31 | m | 49 y | Pso | anti-IL-17A | 7.6 | 17 | 2 | 14 |
| #32 | m | 42 y | Pso | anti-IL-17A | 2.4 | 5 | 1 | 0 |
| #33 | m | 53 y | Pso/PsoA | anti-IL-17A | 0 | 3 | 0 | 0 |
| #34 | f | 70 y | Pso | anti-IL-17A | 23 | 3.8 | 4.4 | 19 |
| #35 | m | 75 y | Pso | anti-IL-17A | 0 | 0 | 0 | 0 |
| #36 | f | 66 y | Pso/PsoA | anti-IL-17A | 2 | 9 | 2 | 0 |
| #37 | m | 59y | Pso | anti-TNFα | 15.9 | 25 | 4 | 3 |
| #38 | m | 56 y | Pso | anti-IL-23 | 10.5 | 17 | 0 | 1 |
| #39 | m | 20 y | Pso | anti-IL-23 | 3.3 | 11 | 6.5 | 0 |
| #40 | m | 52 y | Pso | anti-IL-23 | 5.2 | 3 | 1 | 1 |
| #41 | m | 55y | Pso | anti-IL-23 | 17.2 | 24 | 11 | 8 |
| #42 | m | 31 y | Pso | anti-IL-23 | 1 | 2 | 1 | 0 |
| #43 | m | 62 y | Pso | anti-IL-23 | 0 | 4 | 0 | 0 |
| #44 | f | 47y | Pso | anti-IL-23 | 2 | 1 | 1 | 1 |
| #45 | m | 58 y | Pso | anti-IL-23 | 2.4 | 1 | 1 | 0 |
| #46 | m | 57 y | Pso | anti-IL-23 | 0.6 | 7 | 0 | 6 |
| #47 | m | 65 y | Pso | anti-IL-23 | 3.3 | 1 | 1 | 0 |
| #48 | f | 57 y | Pso | anti-IL-23 | 20 | 28 | 1 | 1 |
| #49 | m | 58 y | Pso | anti-IL-23 | 1 | 14 | 0 | 25 |
| #50 | m | 23 y | Pso | anti-IL-23 | 2 | 1 | 2 | 0 |
| #51 | f | 56 y | Pso | anti-IL-23 | 0.9 | 3 | 1 | 0 |
| #52 | m | 40 y | Pso | anti-IL-23 | 3 | 2 | 2 | 1 |
| #53 | m | 64y | Pso | anti-IL-23 | 4 | 5 | 1.6 | 1 |
| #54 | f | 45 y | Pso | anti-IL-23 | 4 | 2 | 2 | 1 |
| #55 | m | 59 y | Pso | anti-IL-23 | 2 | 2 | 1 | 0 |
| #56 | m | 60 y | Pso | anti-IL-23 | 6 | 3 | 2 | 1 |
| #57 | m | 67 y | Pso | anti-IL-23 | 2 | 0 | 1 | 0 |
| #58 | m | 37 y | Pso | anti-IL-23 | 5 | 3 | 2 | 2 |
| #59 | m | 56 y | Pso | anti-IL-23 | 0.8 | 4 | 0 | 3 |
| #60 | f | 38 y | Pso | anti-IL-23 | 10 | 0 | 0 | 0 |
| #61 | m | 63 y | Pso | anti-IL-23 | 6 | 0 | 3 | 0 |
| #62 | f | 35 y | Pso | anti-IL-23 | 6 | 15 | 0 | 0 |
| #63 | m | 67 y | Pso/PsoA | anti-TNFα | 0 | 0 | 0 | 0 |
| #64 | m | 67 y | Pso | anti-TNFα | 0 | 0 | 1 | 0 |
| #65 | m | 30 y | Pso | anti-IL-12/23 | 2.3 | 15 | 1 | 17 |
| #66 | m | 59 y | Pso/PsoA | anti-IL-12/23 | 0 | 0 | 0 | 5 |
| #67 | m | 57 y | Pso | anti-IL-12/23 | 0 | 0 | 0 | 0 |
| #68 | m | 53 y | Pso | anti-IL-12/23 | 1 | 1 | 1 | 0 |
| #69 | m | 36 y | Pso | anti-IL-12/23 | 1 | 0 | 1 | 1 |
| #70 | f | 84 y | Pso | anti-IL-12/23 | 0 | 7 | 0 | 3 |
| #71 | f | 30 y | Pso | anti-IL-12/23 | 1.6 | 2 | 0 | 2 |
| #72 | m | 38 y | Pso | anti-IL-12/23 | 1.6 | 2 | 1 | 1 |
| #73 | f | 49 y | Pso/PsoA | anti-IL-12/23 | 1.1 | 3 | 1 | 2 |
| #74 | m | 42 y | Pso/PsoA | anti-IL-12/23 | 4 | 18 | 2 | 15 |
| #75 | m | 74 y | Pso/PsoA | anti-IL-12/23 | 0.6 | 1 | 0.9 | 1 |
| #76 | m | 77 y | Pso | anti-IL-12/23 | 0 | 2 | 0 | 10 |
| #77 | f | 48 y | Pso | anti-IL-12/23 | 2 | 1 | 1,2 | 0 |
| #78 | m | 69 y | Pso/PsoA | anti-IL-12/23 | 0 | 1 | 0 | 1 |
| #79 | f | 46 y | Pso | anti-IL-12/23 | 0 | 1 | 0 | 0 |
| #80 | m | 84 y | Pso | anti-IL-12/23 | 0 | 1 | 0 | 1 |
| #81 | m | 67 y | Pso | anti-IL-12/23 | 0 | 3 | 0 | 4 |
| #82 | f | 54 y | Pso | anti-IL-12/23 | 0 | 2 | 0 | 1 |
| #83 | m | 47y | Pso | anti-IL-12/23 | 1 | 0 | 0,6 | 0 |
| #84 | m | 51 y | Pso/PsoA | anti-IL-12/23 | 0.6 | 0 | 0 | 0 |
| #85 | m | 48 y | Pso/PsoA | anti-IL-12/23 | 1 | 1 | 1 | 1 |
| #86 | m | 60 y | Pso/PsoA | anti-IL-12/23 | 0 | 0 | 0 | 0 |
| #87 | m | 50 y | Pso | anti-IL-12/23 | 3.2 | 0 | 1 | 0 |
| #88 | f | 58 y | Pso/PsoA | anti-IL-12/23 | 1 | 10 | 1 | 6 |
| #89 | m | 55 y | Pso | anti-IL-12/23 | 1 | 9 | 1 | 0 |
| #90 | m | 62 y | Pso | anti-IL-23 | 1.2 | 22 | 0 | 0 |
| #91 | f | 53 y | Pso | anti-IL-23 | 10.2 | 15 | n.a. | 1 |
| #92 | m | 52 y | Pso | anti-IL-23 | 8.9 | 3 | 4 | 2 |
| #93 | m | 57 y | Pso | anti-IL-23 | 0 | 0 | 1,6 | 0 |
| #94 | m | 64 y | Pso | anti-IL-23 | 5.8 | 18 | 1 | 1 |
| #95 | f | 38 y | Pso | anti-IL-23 | 1.6 | 10 | 0 | 0 |
| #96 | m | 53 y | Pso | anti-IL-23 | 11.8 | 11 | 0 | 2 |
| #97 | m | 23 y | Pso | anti-IL-23 | 12 | 12 | n.a. | 6 |
| #98 | f | 63 y | Pso | anti-IL-23 | 5.8 | 25 | 1 | 5 |
| #99 | m | 53 y | Pso | anti-IL-23 | 1 | 6 | 1 | 2 |
| #100 | m | 43 y | Pso | anti-IL-23 | 5.6 | 14 | 4 | 2 |
| #101 | f | 66 y | Pso | anit-IL-23 | 0 | 2 | 0 | 1 |
| #102 | f | 25 y | Pso | anti-IL-23 | 1,2 | 1 | 1 | 1 |
| #103 | m | 18y | Pso | anit-IL-23 | 42.7 | 12 | 4 | 0 |

Abbreviation: anti-TNFα, tumour necrosis factor α inhibitor; DLQI, Dermatology Life Quality Index; IL, Interleukin; n, number; PASI, Psoriasis Area and Severity Index; y, years; n.a., not applicable; Pso, Psoriasis; PsoA, Psoriatic arthritis.
