# Supplementary material for: Paradoxical improvement of life quality in the COVID-19 era in psoriasis patients
Source: PLoS One. 2022 Sep 27;17(9):e0275293. doi: 10.1371/journal.pone.0275293 (PMC9514635; doi:10.1371/journal.pone.0275293)
Supplement: S2 Table — (DOCX) [file pone.0275293.s002.docx]

**S2 Table. Dermatology Life Quality Index measurement of social activities during COVID-19 lockdown**

| **Patient ID** | **Shopping, looking after home/garden** | **Social/Leisure activities** | **Sport** | **Working/Studying** | **Interaction (partner, close friends, relatives)** |
| --- | --- | --- | --- | --- | --- |
| #1 | not at all | not at all | not at all | not relevant | not relevant |
| #2 | not at all | not at all | not at all | not at all | not at all |
| #3 | not at all | not at all | not at all | a little | not relevant |
| #4 | not relevant | a lot | not relevant | not relevant | not relevant |
| #5 | not at all | not at all | not at all | not at all | not at all |
| #6 | not at all | not at all | not at all | not at all | not at all |
| #7 | not at all | not at all | not at all | not at all | not at all |
| #8 | not relevant | not relevant | not relevant | not relevant | not relevant |
| #9 | not relevant | not relevant | not relevant | not relevant | not relevant |
| #10 | not at all | not at all | a little | not relevant | not relevant |
| #11 | not at all | a lot | a little | a little | not relevant |
| #12 | not at all | not at all | not at all | not at all | not at all |
| #13 | not relevant | not relevant | not at all | not at all | not relevant |
| #14 | not at all | not at all | not at all | not at all | not at all |
| #15 | not at all | not at all | not at all | not relevant | not relevant |
| #16 | not at all | a lot | not at all | not at all | not at all |
| #17 | not relevant | not relevant | not relevant | not relevant | not relevant |
| #18 | not relevant | not relevant | not relevant | not relevant | not relevant |
| #19 | not relevant | not relevant | not relevant | not relevant | not relevant |
| #20 | not at all | not at all | not at all | not at all | not at all |
| #21 | not at all | not at all | not at all | not at all | not at all |
| #22 | not relevant | not relevant | not relevant | not at all | not relevant |
| #23 | not at all | a lot | not at all | not at all | not at all |
| #24 | not relevant | not relevant | not relevant | not relevant | not relevant |
| #25 | not at all | not at all | not at all | not at all | not at all |
| #26 | not at all | a little | a little | not at all | not at all |
| #27 | not relevant | not relevant | not relevant | not relevant | not relevant |
| #28 | not at all | not at all | not at all | not at all | not relevant |
| #29 | not at all | a little | not at all | not at all | not at all |
| #30 | not relevant | not relevant | not relevant | not relevant | not relevant |
| #31 | not at all | not at all | not at all | a little | not relevant |
| #32 | not at all | not relevant | not relevant | not relevant | not relevant |
| #33 | not relevant | not relevant | not relevant | not relevant | not relevant |
| #34 | not at all | a lot | a little | a little | not relevant |
| #35 | not at all | not relevant | not relevant | not relevant | not relevant |
| #36 | not at all | a little | not at all | not at all | not at all |
| #37 | not at all | not relevant | not relevant | not relevant | not relevant |
| #38 | not at all | not at all | not at all | not at all | not relevant |
| #39 | not relevant | not relevant | not relevant | not relevant | not relevant |
| #40 | not at all | not relevant | not relevant | not relevant | not relevant |
| #41 | not at all | a lot | a little | a little | not at all |
| #42 | not at all | not relevant | not relevant | not relevant | not relevant |
| #43 | not relevant | a lot | not at all | not at all | not relevant |
| #44 | not relevant | not relevant | not relevant | not relevant | not relevant |
| #45 | not at all | not relevant | not relevant | not relevant | not relevant |
| #46 | not relevant | a little | a little | a little | not at all |
| #47 | not at all | not relevant | not relevant | not relevant | not relevant |
| #48 | not at all | not relevant | not relevant | not relevant | not relevant |
| #49 | not at all | a lot | not at all | not at all | not at all |
| #50 | not relevant | not relevant | not relevant | not relevant | not relevant |
| #51 | not relevant | a little | not at all | not at all | not at all |
| #52 | not at all | not relevant | not relevant | not relevant | not relevant |
| #53 | not at all | not relevant | not relevant | not relevant | not relevant |
| #54 | not relevant | not relevant | not relevant | not relevant | not relevant |
| #55 | not relevant | not relevant | not relevant | not relevant | not relevant |
| #56 | not at all | not relevant | not at all | not at all | not at all |
| #57 | not at all | not relevant | not at all | not at all | not at all |
| #58 | not relevant | a lot | not relevant | not relevant | not relevant |
| #59 | not at all | not relevant | not at all | not at all | not at all |
| #60 | not at all | not relevant | not at all | not at all | not relevant |
| #61 | not relevant | a little | not relevant | not relevant | not relevant |
| #62 | not at all | not relevant | not at all | not at all | not at all |
| #63 | not relevant | not relevant | not relevant | not relevant | not relevant |
| #64 | not at all | not relevant | not relevant | not relevant | not relevant |
| #65 | not relevant | a lot | a little | a little | not relevant |
| #66 | not at all | not relevant | not relevant | not relevant | not relevant |
| #67 | not relevant | not relevant | not relevant | not relevant | not relevant |
| #68 | not at all | not relevant | not relevant | not relevant | not relevant |
| #69 | not at all | not relevant | not relevant | not relevant | not relevant |
| #70 | not relevant | a lot | not at all | not at all | not at all |
| #71 | not at all | not relevant | not at all | not at all | not at all |
| #72 | not relevant | not relevant | not relevant | not relevant | not relevant |
| #73 | not at all | not at all | not at all | not at all | not at all |
| #74 | not at all | a little | not at all | a little | not at all |
| #75 | not at all | not at all | not at all | not relevant | not relevant |
| #76 | not relevant | a lot | not relevant | a little | not relevant |
| #77 | not at all | not at all | not at all | not at all | not at all |
| #78 | not relevant | a lot | not relevant | not relevant | not relevant |
| #79 | not relevant | not relevant | not relevant | not relevant | not relevant |
| #80 | not relevant | not relevant | not relevant | not relevant | not relevant |
| #81 | not relevant | not relevant | not at all | not at all | not at all |
| #82 | not at all | a little | not at all | not at all | not at all |
| #83 | not at all | not at all | not at all | not at all | not at all |
| #84 | not relevant | not relevant | not at all | not at all | not at all |
| #85 | not at all | not at all | not at all | not at all | not at all |
| #86 | not at all | not at all | not at all | not relevant | not relevant |
| #87 | not at all | not at all | not at all | not at all | not relevant |
| #88 | not relevant | a lot | not at all | not relevant | not relevant |
| #89 | not at all | not at all | not at all | not at all | not at all |
| #90 | not at all | not at all | not at all | not at all | not at all |
| #91 | not at all | not at all | not at all | not at all | not relevant |
| #92 | not relevant | not relevant | not at all | a little | not relevant |
| #93 | not at all | a little | not at all | not at all | not at all |
| #94 | not at all | not at all | not at all | not at all | not at all |
| #95 | not at all | not at all | not at all | not at all | not at all |
| #96 | not at all | not at all | not at all | not relevant | not relevant |
| #97 | not relevant | not relevant | not at all | not at all | not at all |
| #98 | not at all | a little | not at all | not at all | not at all |
| #99 | not relevant | not relevant | not at all | a little | not relevant |
| #100 | not relevant | a lot | not at all | not at all | not at all |
| #101 | not at all | not at all | not at all | not at all | not at all |
| #102 | not relevant | not relevant | not at all | not relevant | not relevant |
| #103 | not at all | not at all | not at all | not at all | not relevant |
